# Comprehensive insights into the prescribing trends of carbamazepine, lamotrigine, lithium, and valproate in the UK Primary Care from 1995 to 2018

**DOI:** 10.1371/journal.pone.0351169

**Published:** 2026-06-17

**Authors:** Siti Watiqah Samsuddin, Juan Carlos Bazo-Alvarez, Patricia Schartau, Irene Petersen

**Affiliations:** 1 Department of Primary Care and Population Health, Institute of Epidemiology and Health Care, University College London, London, United Kingdom; 2 Escuela de Psicología, Universidad César Vallejo, Trujillo, Peru; 3 Centre for Primary Care, University Hospital Regensburg & University of Regensburg, Regensburg, Bavaria, Germany; 4 Department of Clinical Epidemiology, Aarhus University, Aarhus, Denmark; University of Diyala College of Medicine, IRAQ

## Abstract

**Background:**

Mood stabilisers carbamazepine, lamotrigine and valproate remain the cornerstone medication in the treatment of epilepsy, and in the management of psychiatric disorders alongside lithium. Prescribing decisions are influenced by patient’s preference, clinical history, and risk. This study examines mood stabiliser initiation and prevalence prescribing trends in UK primary care (1995–2018), stratified by sex (male and females) and age (18–39, 40–59, 60–79 and 80–99 years), irrespective of diagnosis.

**Method:**

Cohort studies using UK anonymised electronic primary care data from IQVIA Medical Research Data (IMRD) of individuals aged 18–99 years registered with participating General Practice.

**Results:**

Carbamazepine prescribing declined sharply across ages and sexes since the early 2000s. In contrast, lamotrigine prescribing increased markedly from 2005, thus becoming most initiated among individuals aged 18–39 years by 2018. Carbamazepine initiation predominated in individuals aged 40–99 years, and more so in females than males. Valproate initiation decreased throughout study period: (2018 vs 1995) adjusted Incidence Rate Ratio (aIRR) ranged from 0.12–0.44. However, prevalence rose in males (18–79 years) with adjusted Prevalence Rate Ratio (aPRR) ranged from 1.14–1.60, and females (40–79 years) at aPRR 1.12–1.31. By 2018, valproate was still prevalent among 18–39 years males (3.40/1000 individuals [3.19–3.61]) compared to females (1.18/1000 individuals [1.06–1.30]). Carbamazepine, lamotrigine and valproate were almost equally prescribed by 2018, while lithium the least prescribed. Mood stabiliser prescribing was higher among socioeconomically deprived individuals, both in terms of initiation and prevalence: (Townsend 5 vs Townsend 1) aIRR ranged from 1.07–3.26, and aPRR 1.17–2.90.

**Conclusion:**

Over the study period across demographics, carbamazepine prescribing declined while lamotrigine prescribing increased substantially. By 2018, carbamazepine was mainly initiated in males and females aged 40–99 years and lamotrigine dominated in 18–39 years. Valproate initiation fell but remained prevalent in 18–39 years males; lithium prescribing remained low. Mood stabilisers were frequently prescribed in socioeconomically deprived individuals.

## Introduction

Anticonvulsants such as carbamazepine, lamotrigine and valproate, which are essentially prescribed for epilepsy, and lithium, have been prescribed as mood stabilisers for the treatment of bipolar disorder. In some instances, these medications may be prescribed as adjunctive treatment for other psychiatric disorders, which might be off label prescribing if supported by sufficient evidence for their effectiveness despite not being within the medication’s marketing authorisation [1]. For example, lithium is approved for management of treatment-resistant depression whereas lamotrigine can be used as off-label augmentation treatment for depression and treatment-resistant schizophrenia [2,3]. The choice of anticonvulsant/mood stabiliser prescribed often involves patients’ preferences based on tolerance to any associated side-effects and commitment to monitoring requirement, and the clinician’s assessment of risks and benefits guided by patients’ clinical histories. Additionally, it is essential to consider the seizure type and epilepsy syndrome when prescribing anticonvulsants for epilepsy [4]. Valproate is not recommended for females of childbearing age (55 years and younger) except when the benefits justify the potential risks and a pregnancy prevention programme is in place [5]. Given the narrow therapeutic window of lithium, treatment with lithium therefore requires regular blood tests including monitoring for kidney function [5]. While this demands additional effort, this also represents an advantage for both patients and clinicians in managing its effectiveness and side effects [6]. Over the past decades, previous research on mood stabilisers prescribing has primarily focused on individuals diagnosed with bipolar disorder. There are limited studies that reported anticonvulsant prescribing trends among populations other than children or women of childbearing age. Given the broad indication of carbamazepine, lamotrigine, lithium and valproate, our study offers a more comprehensive analysis of their prescribing trends over an extended period, independent of diagnosis.

## Method

### Study design

This study comprises of a series of cohort studies conducted from 1 January 1995–31 December 2018.

### Setting

In the UK healthcare system, primary care providers, particularly general practitioners (GPs), serve as the initial point of contact for patients. When needed, GPs may refer individuals for more specialised treatments. While treatment may be started outside primary care, it is typically managed long-term by the GPs [5]. Prescriptions within primary care are systematically coded using the Anatomical Therapeutic Classification (ATC) and British National Formulary (BNF) standards, aligned with NHS England’s Dictionary of Medicines and Devices [7].

### Data source

Anonymised electronic primary care records were sourced from the IQVIA Medical Research Database (IMRD), incorporating THIN, a Cegedim database. IMRD includes information from over 790 general practices across the UK, representing approximately 6% of the national population. Thus, providing a demographically and geographically representative sample of the UK population [8].

IMRD contains individual-level data including sex, year of birth, date of death, diagnoses and dates of diagnoses, and social deprivation (Townsend score). Additional data incorporated in the database includes information such as individuals’ date of registration and transfer with the general practice, prescribed medications including generic drug name, formulation, and the dates prescriptions were issued.

The data’s reliability was assessed using two key markers: acceptable mortality rate (AMR) and acceptable computer usage (ACU). ACU identifies the point at which a practice began recording, on average, at least two therapy entries, one medical entry, and one additional health record per patient each year, indicating a shift from paper-based to digital recordkeeping [9]. AMR is defined as the date when a practice’s mortality records were aligned with the general UK population, adjusted for the age and sex distribution of its population [10].

Socioeconomic status was represented by Townsend scores, with a scale from 1 (least deprived) to 5 (most deprived). These scores are derived from census data based on four indicators of deprivation: non-home ownership, non-car ownership, unemployment and overcrowding [11].

### Participants

The study population included all individuals aged from 18 to 99 who were registered with participating GPs at any time during the study period.

### Treatment initiation denominator

Follow-up commenced from the latest of the following: the study’s start date (1 January 1995), the individual’s 18^th^ birthday, registration with a participating practice, or the date a practice began contributing data of high quality (ACU and AMR date). Follow-up concluded at the earliest of these: the study end date (31 December 2018), the individual’s death, de-registration from the participating practice, or for the study of initiation, the date of the first recorded mood stabiliser. We did not have access to data beyond 2018.

### Treatment prevalence denominator

For the prevalence denominator, only time periods where individuals contributed data for the entire calendar year were considered. For example, if an individual’s registration and de-registration dates were 17 July 1997 and 10 June 2003 respectively, data from 1 January 1998–31 December 2002, would be included for that individual.

### Definition of initiation and prevalence

Some individuals may be prescribed mood stabiliser several times in their lives. To define treatment initiation, individuals prescribed mood stabiliser were considered to have started a new treatment if there was a gap of more than 12 months between two consecutive prescriptions. Prevalence was calculated as the number of individuals who were prescribed at least one mood stabiliser within each calendar year of the study period. For instance, an individual prescribed mood stabiliser from February to July 2002, and again from June to October 2008, would contribute to the prevalence data for both 2002 and 2008.

### Treatments considered

The four mood stabilisers examined in this study were carbamazepine, lamotrigine, valproate, and lithium.

### Statistical analysis

Unadjusted initiation rates were expressed per 1,000 person-years at risk (PYAR), and unadjusted prevalence was calculated as the number of individuals receiving at least one mood stabiliser medication (numerator), divided by the total number of individuals registered with a GP in a given year (denominator). All initiation and prevalence estimates were stratified by sex (male and female), and age groups (18–39, 40–59, 60–79 and 80–99 years). All estimates reported by calendar year, and socioeconomic deprivation level based on Townsend quintiles (with 1 representing the least deprived and 5 the most deprived).

To characterise the demographic profile of study participants, the analysis focused on individuals who were registered in 1995 and/or 2018. Thus, representing the start and end of the study period. Additional demographic details are available in S1 Table.

Multivariable Poisson regression models were fitted to assess changes in initiation and prevalence over the calendar years, reported as Incidence Rate Ratios (IRR) and Prevalence Rate Ratios (PRR), respectively, with 1995 serving as the reference year. The IRR and PRR estimates were stratified by sex and age groups and reported with 95% confidence intervals. While all eligible individuals were included in the study, stratified analyses were limited by data availability. For lamotrigine, data are reported from 2005 onwards, with that year serving as the reference point. For lithium, individuals aged 80–99 years were not reported in the stratification analysis due to insufficient data across the study period. Socioeconomic deprivations were examined using Townsend score 1 as the reference, with other covariates (calendar year) adjusted accordingly. All analyses were carried out using Stata software version 18.5 for Windows (StataCorp, College Station, TX, United States).

### Ethics

IMRD has NHS Health Research Authority (NHS Research Ethics Committee ref 23/EM/0151) generic approval for medical research and treatment analysis. Scientific approval for this study was obtained from IMRD’s Scientific Review Committee (SRC) in February 2022 (SRC reference number: 22SRC009). As this research utilises anonymised routinely collected data, no institutional approval was needed. Additionally, no direct contact with individuals nor consent were required. Anonymised data for the purpose of research were accessed between 1 March 2022 and 30 December 2024, and they were treated with strict confidentiality.

## Results

### Demographics of participants

The study included 408,184 individuals in 1995, increasing to 1,927,626 individuals by 2018, with females comprising slightly over 50% of the sample. Individuals aged 18–39 years constituted 36.7% of the study in 1995, declining slightly to 32.4% by 2018. On the other hand, the proportion of individuals aged 40–59, 60–79 and 80–99 years increased from 34.5%, 23.0% and 5.6% in 1995, to 35.1%, 24.9% and 7.6% in 2018, respectively (S1 Table). The proportion of individuals in the study population from least deprived socioeconomic background (Townsend score: 1) dropped from 28.7% in 1995 to 21.4% in 2018. In contrast, the proportion of individuals from more deprived background (Townsend score: 5) rose from 12.6% in 1995 to 14.3% in 2018.

**Fig 1:**
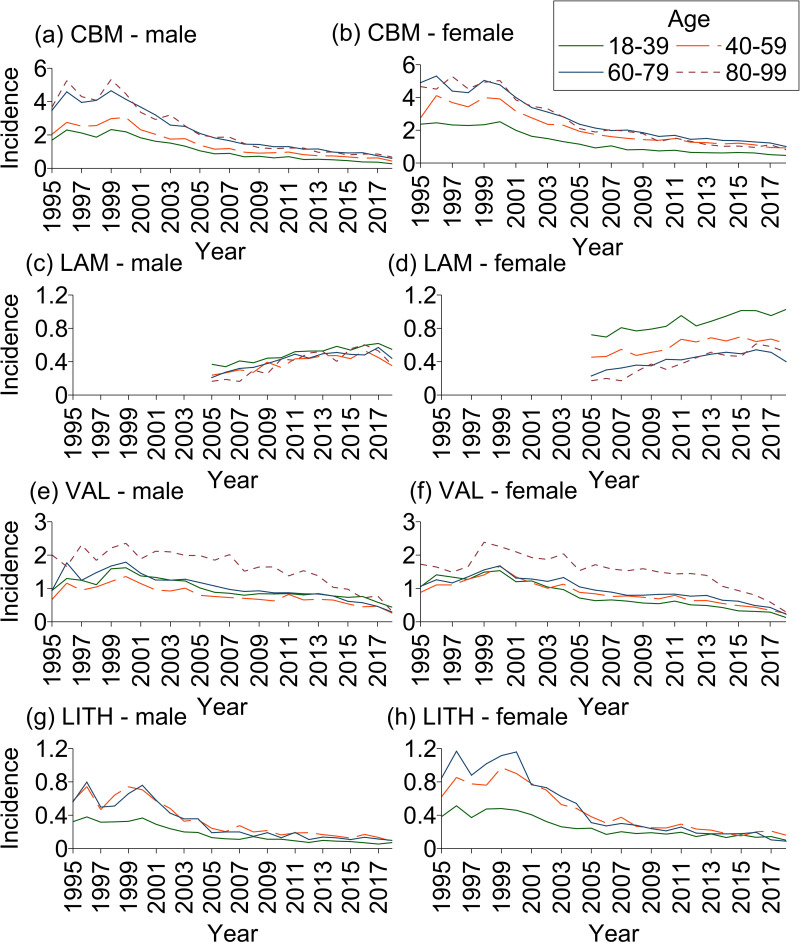
Mood stabilisers initiation per 1,000 PYAR across age groups, stratified by sex (1995 - 2018). Panels show prescribing trends for carbamazepine **(a-b)**, lamotrigine **(c-d)**, valproate **(e-f)** and lithium **(g-h)**.

**Fig 2:**
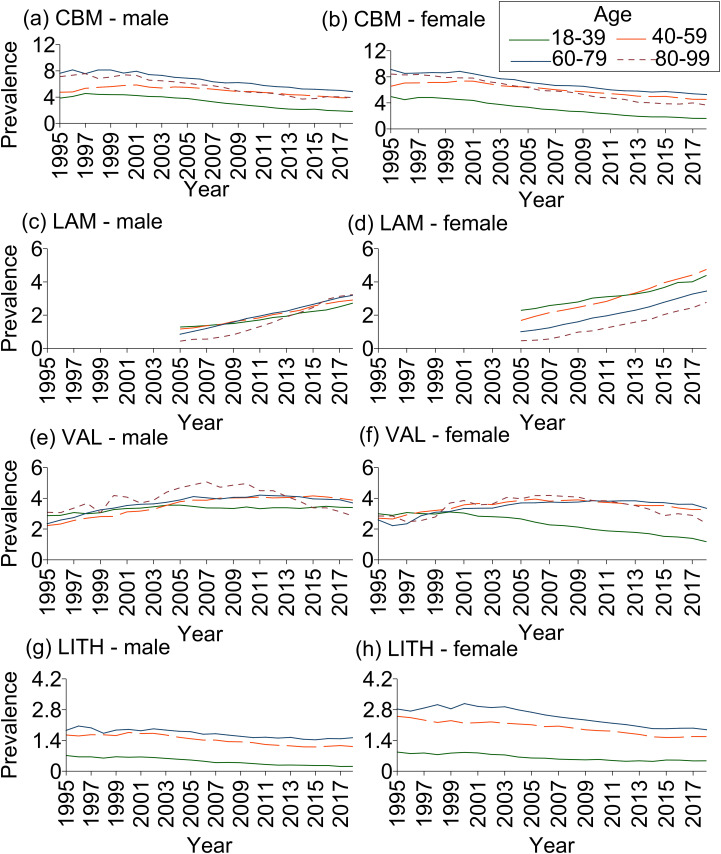
Mood stabilisers prevalence per 1,000 individuals across age groups, stratified by sex (1995 - 2018). Panels show prescribing trends for carbamazepine **(a-b)**, lamotrigine **(c-d)**, valproate **(e-f)** and lithium **(g-h)**.

Figs 1 and 2 in our study described the unadjusted initiation and unadjusted prevalence of mood stabiliser (carbamazepine, lamotrigine, valproate and lithium) prescribing by age groups (18–39, 40–59, 60–79 and 80–99 years) from 1995 to 2018, stratified by sex (male and female). Due to limited data availability, lamotrigine data is only reported from 2005 onwards, and data for individuals aged 80–99 years prescribed lithium is not included.

1 **Initiation and prevalence of mood stabilisers over the study period.**

### Carbamazepine

The initiation of carbamazepine remained relatively stable across all demographics between 1995 and 2000, followed by a substantial decline thereafter (Fig 1a–1b). By 2018, the overall crude initiation rates among males were 0.27/1000 PYAR (18−39 years), 0.44/1000 PYAR (40−59 years), 0.60/1000 PYAR (60−79 years), and 0.67/1000 PYAR (80−99 years) (adjusted Incidence Rate Ratios are provided in S2 File). Among females, the declines were also substantial: by 2018, the initiation rates were 0.47/1000 PYAR (18−39 years), 0.92/1000 PYAR (40−59 years), 1.01/1000 PYAR (60−79 years), and 0.81/1000 PYAR (80−99 years) (S2 File). Thus, females aged above 40 years were more likely than males to be initiated carbamazepine: (males vs females) aIRR 1.47 [1.43–1.50] in 40−59 years; aIRR 1.21 [1.18–1.24] in 60−79 years; aIRR 1.13 [1.07–1.18].

Likewise, carbamazepine prevalence dropped (Fig 2a–2b). Among individuals aged 18–59 years, prevalence dropped earlier in females (between 1999 and 2001) compared to males (2005). By 2018, prevalence had dropped by 55% (1.80/1000 individuals) in males aged 18–39 years, and 25% among 40–59 years (3.87/1000 individuals); prevalence dropped 69% in females 18–39 years (1.60/1000 individuals) and 35% in 40–59 years (4.53/1000 individuals) (adjusted Prevalence Rate Ratios are provided in S2 File). In individuals aged 60–99 years, prevalence started to decline after 2001/2002. By 2018, among those aged 60–79 years, the prevalence were 4.84/1000 individuals in males (36% reduction) and 5.27/1000 individuals in females (41% reduction). Among those aged 80–99 years, the prevalence were 3.93/1000 individuals in males (45% reduction) and 3.66/1000 individuals in females (56% reduction) (S2 File).

### Lamotrigine

The initiation and prevalence of lamotrigine demonstrated a gradual increase from 2005 to 2018 among both males and females across all demographics (Fig 1c–1d). Although lamotrigine initiation was the highest in the younger age (18–39 years) by 2018, the relative rate of increase was steeper in older individuals (60–99 years) (Fig 1c–1d and S3 File). Among 18–39 years, the crude initiation of lamotrigine was 0.55/1000 PYAR (45% increase) in males, and 1.03/1000 PYAR in females (38% increase). In the same year, the initiation increased by 39% (0.35/1000 PYAR), and 28% (0.62/1000) PYAR in males and females aged 40–59 years, respectively. In contrast, the initiation doubled since 2005 in males aged 60–79 years (0.44/1000 PYAR), and 74% increase (0.40/1000 PYAR) in females of the same age group (Fig 1c–1d and S3 File). While among those aged 80–99 years, by 2018 the initiation doubled in males (0.16 to 0.36/1000 PYAR) and tripled in females (0.17 to 0.52/1000 PYAR).

In terms of prevalence, by 2018 the crude prevalence doubled in males aged 18–39 years (1.29 to 2.72/1000 individuals) and 86% increase in females (2.29 to 4.39/1000 individuals) (Fig 2c–2d and S3 File). Prevalence increased by at least two-fold in males and females aged 40–59 years: 1.17 to 2.91/1000 individuals (males); 1.68 to 4.75/1000 individuals (females). In the same period, the increase was at least three-fold in males and females aged 60–79 years: 0.86 to 3.19/1000 individuals (males); 1.00 to 3.45/1000 individuals (females). The prevalence increase was greatest in individuals aged 80–99 years: at least seven-fold in males (0.44 to 3.23/1000 individuals) and six-fold in females (0.46 to 2.77/individuals) (Fig 2c–2d and S3 File).

### Valproate

The initiation of valproate peaked in 2000 for both males and females for all demographic groups, except females in 80–99 years where initiation peaked in 1999 (Fig 1e–1f and S4 File). By 2018, valproate initiation had greater decline among females aged 18–79 years, compared to their male counterparts (Fig 1e–1f and S4 File). For 18–39 years, 88% reduction in females (1.05 to 0.13/1000 PYAR) was reported vs 56% in males (0.94 to 0.44/1000 PYAR); for 40–59 years, 74% reduction in females (0.88 to 0.25/1000 PYAR) vs 65% in males (0.67 to 0.26/1000 PYAR); for 60–79 years, 78% reduction in females (1.06 to 0.23/1000 PYAR) vs 69% in males (0.94 to 0.29/1000 PYAR). While in individuals aged 80–99 years, valproate declined were similar across both males and females: by 85% (2.01 to 0.31/1000 PYAR), and 83% (1.73 to 0.29/1000 PYAR), respectively (Fig 1e–1f and S4 File).

In terms of valproate prevalence among 18–39 years, contrasting trends were reported: by 2018, valproate prevalence increased by 14% in males (2.87 to 3.40/1000 individuals) but reduced by 63% in females (3.00 to 1.18/1000 individuals). Prevalence increased at greater rate among males aged 40–59 years at 60% (2.22 to 3.88/1000 individuals), compared to 12% for their female counterparts (2.71 to 3.28/1000 individuals). Similarly, those aged 60–79 years, prevalence increase at 58% in males (2.35 to 3.70/1000 individuals) and 31% in females (2.59 to 3.35/1000 individuals). On the other hand, prevalence declined among those aged 80–99 years: at 7% reduction in males, and 17% in females (Fig 2e–2f and S4 File).

## Lithium

The initiation of lithium remained relatively consistent across all demographics from 1995 to 1999/2000, then subsequently declined through to 2018 (Fig 1g–1h). By 2018, the reduction among males and females aged 18–39 years were between 75% and 79%: males (0.32 to 0.07/1000 PYAR) and females (0.38 to 0.10/1000 PYAR) (Fig 1g–1h and S5 File). 86% reduction for males aged 40–59 years (0.57 to 0.09/1000 PYAR) was reported compared to their female counterparts at 76% (0.62 to 0.16/1000 PYAR). Among males and females aged 60–79 years, between 83 and 89% reduction were seen by 2018: males (0.56 to 0.10/1000 PYAR) and females (0.84 to 0.09/1000 PYAR) (Fig 1g–1h and S5 File).

Likewise in the same period, lithium prevalence remained stable in the early 2000, which then declined thereafter (Fig 2g–2h). By 2018, prevalence declined by 70% in males, and 48% in females aged 18–39 years: 0.71 to 0.22/1000 individuals and 0.87 to 0.47/1000 individuals, respectively (Fig 2g–2h and S5 File). Among those aged 40–59 years, the reduction was 36% (1.65 to 1.12/1000 individuals) in males and 41% (2.50 to 1.57/1000 individuals) in females. Decline in prevalence in males and females aged 60–79 years were smaller compared to other age groups: 18% in males (1.86 to 1.53/1000 individuals), and 33% in females (2.83 to 1.89/1000 individuals) (Fig 2g–2h and S5 File).

2 **Comparison between carbamazepine, lamotrigine, valproate and lithium by 2018.**

By 2018, lamotrigine was the commonly initiated mood stabiliser among individuals aged 18–39 years at 0.55/1000 PYAR in males and 1.03/1000 PYAR in females. In contrast, carbamazepine was the predominant mood stabiliser among those aged 40–99 years: males at 0.44/1000 PYAR (40–59 years), 0.60/1000 PYAR (60–79 years), 0.67/1000 PYAR (80–99 years); females at 0.92/1000 PYAR (40–59 years), 1.01/1000 PYAR (60–79 years), 0.81/1000 PYAR (80–99 years). Lithium remained the least frequently initiated mood stabiliser by the end of the study period.

In terms of prevalence by 2018, valproate showed the highest prevalence among males aged 18–59 years: 3.40/1000 individuals (18–39 years) and 3.88/1000 individuals (40–59 years). While among females of the same age groups, lamotrigine predominated: 4.39/1000 individuals (18–39 years) and 4.75/1000 individuals (40–59 years). Among older individuals (60–99 years), carbamazepine remained the dominant mood stabiliser for both sexes. Lithium showed the lowest prevalence by 2018, compared to the other mood stabilisers.

Across all mood stabilisers, the most deprived individuals (Townsend score: 5) consistently experienced the greatest initiation and prevalence, compared to the least deprived individuals (Townsend score: 1), across both age groups and sexes (S2–S5 Files). Specifically, initiation and prevalence in the most affluent areas (Townsend score 1) were nearly half of those seen in the most deprived areas (Townsend score 5) (S2–S5 Files).

## Discussion

### Summary of key findings

This study utilised large primary care data to examine the prescribing patterns of carbamazepine, lamotrigine, lithium and valproate. The key findings of our study are that: 1) Carbamazepine was the most prevalently prescribed among older individuals (40–99 years) throughout the study. However, by 2018, the prescribing of carbamazepine, lamotrigine, and valproate had become nearly equal, while lithium prescribing had notably decreased. 2) Valproate, although initiation declined, it remained prevalently prescribed in younger males (18–39 years) by 2018. 3) Individuals from more deprived socioeconomic backgrounds were more likely to be prescribed mood stabilisers across all demographic groups.

### Comparison with existing literature

Previous studies that predominantly focused on individuals with bipolar disorder, reported a gradual increase of lamotrigine prescribing over the past decades, which is consistent with our findings [12–14]. Studies examining individuals with epilepsy have similarly shown a substantial decrease in carbamazepine and valproate prescribing, alongside a rise in lamotrigine [15]. The increased use of lamotrigine compared to carbamazepine, specifically in younger females, is in line with clinical recommendations [4,5,16], where lamotrigine should be used as an alternative in child-bearing females because of its safety profile; it carries the least risk of congenital malformations when used in pregnancy compared to other mood stabilisers specifically valproate [17]. Relative to other mood stabilisers/anticonvulsants, valproate carries the greatest risk of foetal abnormalities at approximately every 10 out of 100 babies [17]. Prior studies have documented a reduction in valproate prescribing among females of childbearing females [18,19] and in pregnancy [20]. In addition, a substantial decline in conception rates among valproate-exposed women aged 14–45 years has been observed, potentially indicating improved pregnancy planning practices [19]. Despite this decline in valproate initiation across both sexes in our study, valproate remained notably prevalent among younger males. This aligns with previous research indicating a rise in valproate prescribing in males [18,21]. In the past decade, lithium has been markedly underutilised, with prescribing either remained stable or declining [12,13,18,21–23]. Unlike the other anticonvulsants/mood stabiliser, lithium possesses a narrow therapeutic index whereby serum concentrations that may lead to toxicity, while low levels may render the treatment ineffective. Consequently, lithium treatment necessitates regular blood monitoring, particularly during treatment initiation and dose adjustments, which contributes to its reputation as a complex and challenging medication to manage [24].

### Main findings

Carbamazepine prescribing among older individuals, despite its decline and had become nearly on par with lamotrigine and valproate, remained the most prescribed mood stabiliser among older adults (aged 40 years and above) by 2018. Given that carbamazepine is an older-generation anticonvulsant/mood stabiliser, older adults may have been initiated and maintained on this therapy before the introduction of newer medication including lamotrigine. In addition to its psychiatric and antiepileptic indications, carbamazepine remains the only licensed treatment, unless contraindicated, for trigeminal neuralgia, a condition characterised by severe, unilateral facial pain [25]. Thus, wider indications for carbamazepine may have potentially contributed to higher prescribing. However, carbamazepine is a potent enzyme inducer of the cytochrome P450 enzymes which essentially means any other medication prescribed alongside carbamazepine, will be metabolised more rapidly [26]. For instance, co-prescribing of carbamazepine with amlodipine, an antihypertensive medication, may lead to ineffectiveness of amlodipine due to lower plasma concentration of amlodipine than normal resulting from its increased metabolism. In addition, there is evidence that carbamazepine has been linked to hyponatremia, potentially through its antidiuretic action [27]. As older individuals are particularly vulnerable to declining kidney function, the co-administration of other medications with antidiuretic effects can exacerbate complications.

Notwithstanding these effects, carbamazepine remains an effective and valuable mood stabiliser. Therefore, clinical judgement remains crucial weighing the risk and benefit of carbamazepine in older individuals. Regular blood monitoring and assessments of kidney and liver function are essential, and doses should be adjusted where necessary. There is some evidence, although still insufficient, suggesting that long-term carbamazepine treatment may increase the risk of osteopenia, osteoporosis, and fractures by reducing bone mineral density [28,29]. As this population is generally at greater risk, supplementation with vitamin D and calcium may be required. Further study to examine the proportion of co-prescribing of carbamazepine and physical health medications, and therefore their trajectory is encouraged.

By 2018, valproate remained prevalent among younger males in our study. The wider indications for the prescribing of valproate and greater access to medications may have contributed to this high prevalence [14,30]. Valproate has been linked to weight gain [31], thus additional monitoring of weight before and during treatment with valproate is crucial, specifically when valproate is also prescribed alongside weight-affecting medication such as second-generation antipsychotics. There is evidence indicating an association between valproate and a decline in testosterone levels in males with epilepsy, potentially leading to sexual and reproductive dysfunction in this population [32]. Thus, in addition to monitoring weight, it is essential to regularly assess other metabolic factors in younger males, including cholesterol levels. There are concerns around valproate in young males; likewise as observed among child-bearing females, valproate may impact the quality of sperm reversibly and thus fertility [33,34] and has the potential for teratogenic complications. Despite these effects, valproate remains one of the most effective medications for epilepsy and bipolar disorder therefore clinicians should make better judgements when prescribing and de-prescribing weighing the risk and benefits of prescribing valproate [35]. Due to the limitation of our study, which only limits our analysis up to 2018 therefore follow-up studies are highly encouraged.

Carbamazepine, lamotrigine, lithium and valproate are more likely to be prescribed among individuals from socially deprived backgrounds. The potential causes for this are complex and multifactorial. One possible reason that some individuals may be prescribed these medications is due to the clinical indications for their use. For instance, individuals with severe mental illness are more likely to be prescribed mood stabilisers. Severe mental illness may also be associated with socioeconomic status [36,37]. Thus, we see an association between social deprivation and the prescribing of these drugs. However, the association between epilepsy and social deprivation is less clear [38]. Additionally, we see more prescribing among females than males. This may be explained by the fact that females are more likely to be diagnosed with mood disorders whereas males are less likely [21,37]. Moreover, it has been demonstrated than males are less likely to consult with the GPs and therefore may be less likely to receive treatment [39]. By 2018, valproate was more likely to be prescribed among younger males than younger females. This trend may be partly attributed to the known safety profiles of lamotrigine and carbamazepine in minimising the risk of congenital malformations compared to valproate [16,40], making them preferable options for younger females.

The underutilised of lithium have been explored in previous studies [24]. Its suboptimal prescribing may stem from challenges in monitoring and dosing which may also be contributed by the shared care agreements made between some NHS Trusts and GPs. Due to the narrow therapeutic index of lithium and its ability to impair kidney function, any substantial increase in lithium dosage may lead to toxicity [41]. Therefore, it is crucial, specifically in the early treatment, to monitor plasma lithium concentration [5]. Lithium is also known to be associated with weight gain but there is limited evidence that the risk of weight gain with lithium is lower compared to that associated with lamotrigine [42]. Despite this trend, lithium has been reported to be the most effective treatment for bipolar disorder and severe depression [6]. Addressing the underutilisation of lithium and improving monitoring and adherence are essential, with further research needed.

Carbamazepine, lamotrigine and valproate remain the cornerstone medication in the treatment of epilepsy. However, these medications also play a major role in the management of psychiatric disorders, alongside lithium. Lamotrigine and valproate (as sodium valproate or valproic acid) continue to serve as first-line monotherapies for generalised tonic-clonic seizures. For focal seizures and absence seizures, carbamazepine is recommended as a second-line option if first-line treatment prove ineffective [4]. In psychiatry, lithium remains the gold standard for the long-term management of bipolar disorder and treatment-resistant depression. Valproate, particularly in its semi-sodium formulation, is indicated for the treatment of acute mania in bipolar disorder when lithium is contraindicated or poorly tolerated, while other valproate formulations may be used off label. Lamotrigine is licensed for the treatment of bipolar depression, and may be prescribed adjunctively with other anticonvulsant/mood stabiliser to optimise therapeutic outcomes [5]. Overall, given the variability of individual response, tolerability, and safety profiles of these mood stabilisers, it is essential that treatment selection to be tailored to an individual’s needs, preferences and clinical presentation.

### Strengths and limitations

The strength of this study is its reflection of real-world prescribing for mood stabiliser in the UK. While it is likely that initial prescriptions occur in secondary care and are not captured in our data, ongoing treatment is managed in primary care, which is reflected in our data. Due the UK’s nationally funded healthcare system, with minimal private insurer involvement, the datasets used has greater transparency thus supporting the reliability of this study. A limitation of the study is that we do not have access to data beyond 2018 thus limiting our ability to assess trends beyond that point. New guidelines and recommendations have been introduced after 2018. For instance, in 2024, The Medicines and Healthcare Products Regulatory Agency (MHRA) recommends that, not only females under the age of 55 years, but also males, should not be started on valproate unless there is no alternative treatment available and provided that this treatment have been reviewed by two independent specialists [16]. Therefore, future studies to examine the trends of valproate use among these individuals using more recent data are essential.

### Implications for research and practice

Further research on the prescribing of mood stabilisers should particularly focus on 1) the safety profile of valproate in young males and carbamazepine in older individuals, and 2) valproate prescribing trends in young males following the publication of new guidance. Additionally, there is a need for more detailed understanding of the long-term safety and outcomes of co-prescribing mood stabilisers with physical health medications. Finally, further epidemiological studies extending beyond 2018 are necessary to examine emerging trends and clinical practices.

## Conclusion

Over the study period across demographics, carbamazepine prescribing declined while lamotrigine prescribing increased substantially. By 2018, carbamazepine was mainly initiated in males and females aged 40–99 years and lamotrigine dominated in 18–39 years. Valproate initiation fell but remained prevalent in 18–39 years males; lithium prescribing remained low. Mood stabilisers were frequently prescribed in socioeconomically deprived individuals.

**Highlights**

- Carbamazepine initiation predominated in older individuals (40–99 years), particularly among females, and lamotrigine dominated in younger individuals (18–39 years).
- By 2018, carbamazepine, lamotrigine and valproate were almost equally prescribed while lithium remained the least prescribed mood stabiliser.
- Despite a decline in initiation, valproate remains prevalent among younger males aged 18–39 years.
- Individuals from disadvantaged socioeconomic backgrounds are more likely to be prescribed mood stabilisers.

## Supporting information

S1 Table Demographics of the participants in year 1995 and 2018. (PDF)

S2 File Carbamazepine prescribing (1995–2018), stratified by sex and age. a. Initiation, IRR and aIRR by calendar year and social deprivation, stratified by sex among individuals aged 18–39 years, 40–59 years, 60–79 years and 80–99 years. b. Prevalence, PRR and aPRR by calendar year and social deprivation, stratified by sex among individuals aged 18–39 years, 40–59 years, 60–79 years and 80–99 years.
(PDF)

S3 File Lamotrigine prescribing (1995–2018), stratified by sex and age. a. Initiation, IRR and aIRR by calendar year and social deprivation, stratified by sex among individuals aged 18–39 years, 40–59 years, 60–79 years and 80–99 years. b. Prevalence, PRR and aPRR by calendar year and social deprivation, stratified by sex among individuals aged 18–39 years, 40–59 years, 60–79 years and 80–99 years.
(PDF)

S4 File Valproate prescribing (1995–2018), stratified by sex and age. a. Initiation, IRR and aIRR by calendar year and social deprivation, stratified by sex among individuals aged 18–39 years, 40–59 years, 60–79 years and 80–99 years. b. Prevalence, PRR and aPRR by calendar year and social deprivation, stratified by sex among individuals aged 18–39 years, 40–59 years, 60–79 years and 80–99 years.
(PDF)

S5 File Lithium prescribing (1995–2018), stratified by sex and age. a. Initiation, IRR and aIRR by calendar year and social deprivation, stratified by sex among individuals aged 18–39 years, 40–59 years, 60–79 years and 80–99 years. b. Prevalence, PRR and aPRR by calendar year and social deprivation, stratified by sex among individuals aged 18–39 years, 40–59 years, 60–79 years and 80–99 years.
(PDF)
