# Supplementary material for: Comprehensive insights into the prescribing trends of carbamazepine, lamotrigine, lithium, and valproate in the UK Primary Care from 1995 to 2018
Source: PLoS One. 2026 Jun 17;21(6):e0351169. doi: 10.1371/journal.pone.0351169 (PMC13274886; doi:10.1371/journal.pone.0351169)
Supplement: S1 Table — (PDF) [file pone.0351169.s001.pdf]

S1 Table. Demographics of the participants in year 1995 and 2018.

N	1995		2018	
	408,184		1,927,626	
Sex (%)				
Male	197,129	(48.3)	929,357	(48.2)
Female	211,055	(51.7)	998,269	(51.8)
Age band (%)				
18-39	149,951	(36.7)	625,136	(32.4)
40-59	140,712	(34.5)	676,343	(35.1)
60-79	93,936	(23.0)	479,791	(24.9)
80-99	23,585	(5.6)	146,356	(7.6)
Townsend score (%)				
1 (least deprived)	117,011	(28.7)	413,242	(21.4)
2	91,038	(22.3)	422,954	(21.9)
3	78,746	(19.3)	443,569	(23.0)
4	69,903	(17.1)	373,129	(19.4)
5 (most deprived)	51,486	(12.6)	274,732	(14.3)
