# Supplementary material for: Comprehensive insights into the prescribing trends of carbamazepine, lamotrigine, lithium, and valproate in the UK Primary Care from 1995 to 2018
Source: PLoS One. 2026 Jun 17;21(6):e0351169. doi: 10.1371/journal.pone.0351169 (PMC13274886; doi:10.1371/journal.pone.0351169)
Supplement: S5 File — a. Initiation, IRR and aIRR by calendar year and social deprivation, stratified by sex among individuals aged 18–39 years, 40–59 years, 60–79 years and 80–99 years. b. Prevalence, PRR and aPRR by calendar year and social deprivation, stratified by sex among individuals aged 18–39 years, 40–59 years, 60–79 years and 80–99 years. (PDF) [file pone.0351169.s005.pdf]

S5 File. Lithium prescribing (1995-2018), stratified by sex and age.

(a) Initiation, Initiation Rate Ratio (IRR) and adjusted Initiation Rate Ratio (aIRR) by calendar year and social deprivation (Townsend score), stratified by sex among individuals aged:

- 18-39 years
- 40-59 years
- 60-79 years
- 80-99 years

(b) Prevalence, Prevalence Rate Ratio (PRR) and adjusted Prevalence Rate Ratio (aPRR) by calendar year and social deprivation (Townsend score), stratified by sex among individuals aged:

- 18-39 years
- 40-59 years
- 60-79 years
- 80-99 years

(a) Initiation, IRR and aIRR by calendar year and social deprivation, stratified by sex among individuals aged 18-39 years.

	Male						Female					
	18-39 years						18-39 years					
(Year)	I	CI (95%)	IRR	CI (95%)	aIRR	CI (95%)	I	CI (95%)	IRR	CI (95%)	aIRR	CI (95%)
1995	0.32	[0.22,0.46]	1		1		0.38	[0.26,0.53]	1		1	
1996	0.38	[0.27,0.51]	1.17	[0.74,1.87]	1.17	[0.73,1.86]	0.51	[0.39,0.66]	1.34	[0.86,2.10]	1.33	[0.85,2.08]
1997	0.32	[0.23,0.42]	0.98	[0.63,1.54]	0.97	[0.62,1.53]	0.37	[0.28,0.48]	0.97	[0.62,1.52]	0.96	[0.62,1.51]
1998	0.32	[0.24,0.41]	0.99	[0.62,1.59]	0.99	[0.61,1.59]	0.47	[0.38,0.59]	1.24	[0.81,1.90]	1.23	[0.81,1.88]
1999	0.33	[0.26,0.41]	1.01	[0.66,1.55]	1.01	[0.66,1.54]	0.48	[0.40,0.58]	1.26	[0.84,1.88]	1.25	[0.83,1.86]
2000	0.37	[0.30,0.44]	1.13	[0.76,1.70]	1.13	[0.75,1.68]	0.46	[0.39,0.54]	1.20	[0.81,1.79]	1.19	[0.80,1.77]
2001	0.29	[0.24,0.35]	0.90	[0.60,1.35]	0.90	[0.60,1.34]	0.41	[0.35,0.47]	1.06	[0.72,1.58]	1.05	[0.71,1.56]
2002	0.24	[0.20,0.29]	0.74	[0.49,1.10]	0.73	[0.49,1.09]	0.32	[0.28,0.38]	0.85	[0.57,1.26]	0.84	[0.56,1.24]
2003	0.20	[0.16,0.24]	0.62	[0.41,0.93]	0.61	[0.41,0.91]	0.26	[0.22,0.31]	0.68	[0.46,1.02]	0.67	[0.45,1.00]
2004	0.19	[0.16,0.23]	0.60	[0.40,0.89]	0.59	[0.39,0.88]	0.24	[0.20,0.28]	0.63	[0.42,0.93]	0.61	[0.41,0.91]
2005	0.13	[0.10,0.16]	0.41	[0.27,0.62]	0.40	[0.26,0.61]	0.24	[0.21,0.29]	0.64	[0.43,0.95]	0.62	[0.42,0.92]
2006	0.12	[0.09,0.15]	0.36	[0.24,0.56]	0.36	[0.23,0.54]	0.17	[0.14,0.20]	0.44	[0.30,0.66]	0.43	[0.29,0.65]
2007	0.11	[0.09,0.14]	0.34	[0.23,0.53]	0.34	[0.22,0.51]	0.20	[0.17,0.24]	0.53	[0.35,0.79]	0.51	[0.34,0.76]
2008	0.14	[0.12,0.17]	0.44	[0.29,0.67]	0.43	[0.29,0.65]	0.18	[0.15,0.22]	0.47	[0.31,0.71]	0.46	[0.31,0.68]
2009	0.11	[0.09,0.14]	0.35	[0.23,0.53]	0.34	[0.22,0.51]	0.19	[0.16,0.23]	0.50	[0.33,0.74]	0.48	[0.32,0.72]
2010	0.11	[0.09,0.14]	0.35	[0.23,0.53]	0.34	[0.22,0.52]	0.17	[0.14,0.21]	0.46	[0.30,0.68]	0.44	[0.29,0.66]
2011	0.09	[0.07,0.12]	0.29	[0.18,0.44]	0.28	[0.18,0.43]	0.20	[0.16,0.23]	0.51	[0.34,0.76]	0.49	[0.33,0.73]
2012	0.07	[0.06,0.10]	0.23	[0.15,0.36]	0.22	[0.14,0.35]	0.14	[0.12,0.17]	0.38	[0.25,0.56]	0.36	[0.24,0.54]
2013	0.10	[0.08,0.12]	0.30	[0.20,0.47]	0.29	[0.19,0.45]	0.17	[0.15,0.21]	0.46	[0.31,0.68]	0.44	[0.29,0.65]
2014	0.09	[0.07,0.11]	0.27	[0.17,0.42]	0.26	[0.17,0.41]	0.13	[0.11,0.16]	0.35	[0.23,0.53]	0.33	[0.22,0.50]
2015	0.08	[0.06,0.11]	0.26	[0.16,0.41]	0.25	[0.16,0.40]	0.16	[0.13,0.20]	0.43	[0.29,0.65]	0.41	[0.27,0.62]
2016	0.07	[0.05,0.10]	0.22	[0.13,0.36]	0.21	[0.12,0.34]	0.13	[0.10,0.17]	0.35	[0.23,0.54]	0.33	[0.22,0.51]
2017	0.05	[0.03,0.08]	0.17	[0.10,0.30]	0.16	[0.09,0.28]	0.14	[0.11,0.19]	0.38	[0.24,0.59]	0.36	[0.23,0.56]
2018	0.07	[0.05,0.11]	0.22	[0.13,0.38]	0.21	[0.13,0.36]	0.10	[0.07,0.14]	0.26	[0.16,0.43]	0.25	[0.15,0.40]
(Townsend score)												
1	0.12	[0.10,0.13]	1		1		0.17	[0.15,0.18]	1		1	
2	0.13	[0.11,0.14]	1.09	[0.92,1.28]	1.12	[0.95,1.32]	0.19	[0.17,0.21]	1.14	[0.99,1.30]	1.17	[1.02,1.34]
3	0.14	[0.12,0.15]	1.17	[0.99,1.37]	1.24	[1.06,1.46]	0.22	[0.20,0.24]	1.31	[1.15,1.50]	1.39	[1.22,1.58]
4	0.17	[0.15,0.18]	1.42	[1.22,1.66]	1.53	[1.31,1.78]	0.25	[0.23,0.27]	1.50	[1.32,1.70]	1.59	[1.40,1.81]
5	0.20	[0.18,0.22]	1.67	[1.42,1.97]	1.82	[1.55,2.14]	0.31	[0.28,0.34]	1.86	[1.63,2.12]	2.00	[1.76,2.28]

IRR – Incidence Rate Ratio; aIRR – Adjusted Incidence Rate Ratio. Rates were adjusted for other characteristics in this table.

(a) (cont.) Initiation, IRR and aIRR by calendar year and social deprivation, stratified by sex among individuals aged 40-59 years.

	Male						Female					
	40-59 years						40-59 years					
(Year)	I	CI (95%)	IRR	CI (95%)	aIRR	CI (95%)	I	CI (95%)	IRR	CI (95%)	aIRR	CI (95%)
1995	0.57	[0.42,0.76]	1		1		0.62	[0.46,0.82]	1		1	
1996	0.74	[0.59,0.93]	1.30	[0.89,1.90]	1.29	[0.88,1.88]	0.85	[0.68,1.05]	1.37	[0.93,2.03]	1.37	[0.93,2.01]
1997	0.47	[0.36,0.60]	0.82	[0.54,1.22]	0.81	[0.54,1.21]	0.78	[0.63,0.94]	1.25	[0.87,1.79]	1.24	[0.87,1.77]
1998	0.64	[0.53,0.78]	1.12	[0.77,1.64]	1.11	[0.76,1.63]	0.76	[0.63,0.91]	1.22	[0.86,1.75]	1.22	[0.85,1.74]
1999	0.74	[0.63,0.86]	1.29	[0.91,1.84]	1.29	[0.91,1.82]	0.97	[0.84,1.11]	1.56	[1.12,2.18]	1.56	[1.12,2.16]
2000	0.70	[0.61,0.80]	1.23	[0.88,1.72]	1.22	[0.87,1.71]	0.90	[0.80,1.01]	1.45	[1.05,2.00]	1.45	[1.05,1.99]
2001	0.57	[0.50,0.65]	0.99	[0.71,1.40]	0.99	[0.70,1.38]	0.78	[0.69,0.87]	1.26	[0.92,1.72]	1.25	[0.91,1.71]
2002	0.48	[0.42,0.55]	0.84	[0.60,1.18]	0.83	[0.59,1.16]	0.70	[0.63,0.78]	1.13	[0.83,1.55]	1.12	[0.82,1.53]
2003	0.33	[0.28,0.38]	0.57	[0.40,0.81]	0.56	[0.40,0.80]	0.53	[0.47,0.59]	0.85	[0.62,1.16]	0.84	[0.61,1.15]
2004	0.35	[0.30,0.40]	0.61	[0.43,0.86]	0.59	[0.42,0.84]	0.48	[0.43,0.54]	0.78	[0.57,1.06]	0.76	[0.56,1.04]
2005	0.24	[0.21,0.29]	0.43	[0.30,0.61]	0.42	[0.29,0.59]	0.38	[0.33,0.43]	0.62	[0.45,0.85]	0.60	[0.44,0.83]
2006	0.20	[0.17,0.24]	0.36	[0.25,0.51]	0.35	[0.24,0.49]	0.31	[0.27,0.35]	0.49	[0.36,0.68]	0.48	[0.35,0.67]
2007	0.28	[0.24,0.32]	0.48	[0.34,0.68]	0.47	[0.33,0.66]	0.38	[0.33,0.43]	0.60	[0.44,0.83]	0.59	[0.43,0.81]
2008	0.20	[0.17,0.24]	0.35	[0.24,0.50]	0.34	[0.24,0.48]	0.27	[0.23,0.31]	0.43	[0.31,0.60]	0.42	[0.30,0.58]
2009	0.22	[0.18,0.25]	0.38	[0.27,0.54]	0.37	[0.26,0.52]	0.25	[0.21,0.29]	0.40	[0.29,0.56]	0.39	[0.28,0.54]
2010	0.16	[0.13,0.20]	0.28	[0.20,0.41]	0.28	[0.19,0.39]	0.25	[0.21,0.29]	0.40	[0.29,0.55]	0.39	[0.28,0.53]
2011	0.19	[0.16,0.22]	0.33	[0.23,0.47]	0.32	[0.22,0.45]	0.29	[0.25,0.34]	0.47	[0.34,0.65]	0.46	[0.33,0.63]
2012	0.19	[0.16,0.22]	0.33	[0.23,0.47]	0.32	[0.22,0.45]	0.23	[0.20,0.27]	0.38	[0.27,0.52]	0.36	[0.26,0.50]
2013	0.17	[0.14,0.20]	0.29	[0.20,0.42]	0.28	[0.19,0.40]	0.22	[0.19,0.26]	0.36	[0.26,0.50]	0.34	[0.25,0.47]
2014	0.15	[0.12,0.18]	0.26	[0.18,0.38]	0.25	[0.17,0.36]	0.18	[0.15,0.22]	0.29	[0.21,0.41]	0.28	[0.20,0.39]
2015	0.13	[0.10,0.16]	0.22	[0.15,0.33]	0.21	[0.14,0.31]	0.15	[0.12,0.19]	0.24	[0.17,0.35]	0.23	[0.16,0.33]
2016	0.17	[0.13,0.21]	0.30	[0.20,0.43]	0.28	[0.19,0.41]	0.20	[0.16,0.25]	0.32	[0.23,0.46]	0.30	[0.22,0.43]
2017	0.13	[0.10,0.18]	0.23	[0.15,0.36]	0.22	[0.15,0.33]	0.21	[0.17,0.26]	0.34	[0.24,0.48]	0.32	[0.22,0.46]
2018	0.09	[0.06,0.12]	0.15	[0.10,0.24]	0.14	[0.09,0.22]	0.16	[0.12,0.21]	0.26	[0.18,0.38]	0.24	[0.17,0.35]
(Townsend score)												
1	0.20	[0.19,0.22]	1		1		0.27	[0.25,0.29]	1		1	
2	0.24	[0.22,0.25]	1.16	[1.03,1.31]	1.21	[1.07,1.36]	0.34	[0.32,0.36]	1.25	[1.13,1.39]	1.30	[1.17,1.44]
3	0.27	[0.25,0.30]	1.35	[1.20,1.53]	1.46	[1.29,1.64]	0.37	[0.34,0.39]	1.35	[1.22,1.50]	1.45	[1.31,1.61]
4	0.33	[0.30,0.35]	1.62	[1.43,1.82]	1.76	[1.56,1.98]	0.44	[0.41,0.47]	1.64	[1.47,1.82]	1.77	[1.60,1.97]
5	0.37	[0.34,0.41]	1.84	[1.62,2.09]	2.03	[1.79,2.31]	0.54	[0.50,0.58]	1.98	[1.76,2.23]	2.19	[1.95,2.46]

IRR – Incidence Rate Ratio; aIRR – Adjusted Incidence Rate Ratio. Rates were adjusted for other characteristics in this table.

(a) (cont.) Initiation, IRR and aIRR by calendar year and social deprivation, stratified by sex among individuals aged 60-79 years.

	Male						Female					
	60-79 years						60-79 years					
(Year)	I	CI (95%)	IRR	CI (95%)	aIRR	CI (95%)	I	CI (95%)	IRR	CI (95%)	aIRR	CI (95%)
1995	0.56	[0.37,0.81]	1		1		0.84	[0.63,1.11]	1		1	
1996	0.80	[0.59,1.05]	1.42	[0.88,2.29]	1.42	[0.88,2.30]	1.17	[0.94,1.44]	1.39	[0.93,2.06]	1.39	[0.93,2.06]
1997	0.50	[0.36,0.67]	0.89	[0.55,1.43]	0.89	[0.55,1.43]	0.88	[0.71,1.08]	1.04	[0.70,1.55]	1.04	[0.70,1.55]
1998	0.51	[0.38,0.67]	0.91	[0.56,1.48]	0.91	[0.56,1.49]	1.02	[0.85,1.21]	1.21	[0.82,1.77]	1.21	[0.82,1.78]
1999	0.66	[0.53,0.81]	1.17	[0.77,1.79]	1.18	[0.77,1.80]	1.11	[0.96,1.29]	1.32	[0.92,1.89]	1.33	[0.93,1.90]
2000	0.76	[0.64,0.89]	1.35	[0.91,2.03]	1.36	[0.91,2.04]	1.16	[1.02,1.31]	1.37	[0.98,1.93]	1.38	[0.98,1.95]
2001	0.58	[0.48,0.69]	1.03	[0.69,1.55]	1.04	[0.69,1.56]	0.77	[0.66,0.88]	0.91	[0.64,1.30]	0.92	[0.64,1.31]
2002	0.42	[0.35,0.51]	0.76	[0.50,1.14]	0.76	[0.50,1.15]	0.73	[0.64,0.83]	0.87	[0.61,1.22]	0.87	[0.62,1.24]
2003	0.36	[0.29,0.43]	0.64	[0.42,0.96]	0.64	[0.43,0.96]	0.62	[0.54,0.71]	0.74	[0.52,1.05]	0.75	[0.53,1.05]
2004	0.36	[0.30,0.43]	0.64	[0.42,0.96]	0.64	[0.43,0.96]	0.54	[0.47,0.62]	0.64	[0.45,0.91]	0.65	[0.46,0.92]
2005	0.19	[0.15,0.24]	0.34	[0.22,0.53]	0.34	[0.22,0.53]	0.31	[0.26,0.37]	0.37	[0.26,0.53]	0.37	[0.26,0.54]
2006	0.20	[0.16,0.25]	0.36	[0.23,0.54]	0.36	[0.23,0.55]	0.27	[0.22,0.33]	0.32	[0.22,0.47]	0.33	[0.23,0.47]
2007	0.20	[0.16,0.25]	0.36	[0.23,0.54]	0.36	[0.24,0.55]	0.30	[0.25,0.36]	0.36	[0.25,0.51]	0.36	[0.25,0.51]
2008	0.15	[0.11,0.19]	0.26	[0.17,0.40]	0.26	[0.17,0.41]	0.28	[0.23,0.33]	0.33	[0.23,0.47]	0.33	[0.23,0.48]
2009	0.19	[0.15,0.24]	0.34	[0.22,0.52]	0.34	[0.22,0.52]	0.24	[0.20,0.29]	0.28	[0.20,0.41]	0.29	[0.20,0.41]
2010	0.13	[0.10,0.17]	0.23	[0.15,0.36]	0.23	[0.15,0.36]	0.21	[0.17,0.26]	0.25	[0.17,0.37]	0.26	[0.18,0.37]
2011	0.19	[0.15,0.24]	0.34	[0.23,0.52]	0.35	[0.23,0.53]	0.26	[0.22,0.31]	0.31	[0.21,0.44]	0.31	[0.22,0.45]
2012	0.11	[0.08,0.14]	0.19	[0.12,0.31]	0.19	[0.12,0.31]	0.18	[0.15,0.23]	0.22	[0.15,0.32]	0.22	[0.15,0.32]
2013	0.14	[0.11,0.18]	0.25	[0.16,0.38]	0.25	[0.16,0.38]	0.18	[0.14,0.22]	0.21	[0.14,0.31]	0.21	[0.14,0.31]
2014	0.13	[0.10,0.17]	0.23	[0.15,0.36]	0.23	[0.15,0.36]	0.18	[0.14,0.22]	0.21	[0.14,0.31]	0.21	[0.14,0.31]
2015	0.11	[0.08,0.15]	0.19	[0.12,0.31]	0.19	[0.12,0.31]	0.18	[0.14,0.23]	0.21	[0.14,0.31]	0.21	[0.15,0.32]
2016	0.14	[0.10,0.18]	0.24	[0.15,0.39]	0.24	[0.15,0.39]	0.19	[0.15,0.25]	0.23	[0.15,0.34]	0.23	[0.15,0.35]
2017	0.11	[0.08,0.16]	0.20	[0.12,0.34]	0.20	[0.12,0.34]	0.10	[0.07,0.15]	0.12	[0.08,0.20]	0.12	[0.08,0.20]
2018	0.10	[0.06,0.14]	0.17	[0.10,0.30]	0.17	[0.10,0.30]	0.09	[0.06,0.13]	0.10	[0.06,0.18]	0.11	[0.06,0.18]
(Townsend score)												
1	0.22	[0.20,0.24]	1		1		0.31	[0.29,0.33]	1		1	
2	0.22	[0.20,0.25]	1.01	[0.87,1.17]	1.03	[0.89,1.19]	0.37	[0.34,0.40]	1.20	[1.06,1.36]	1.22	[1.08,1.37]
3	0.23	[0.21,0.26]	1.05	[0.90,1.22]	1.07	[0.92,1.24]	0.36	[0.33,0.39]	1.17	[1.03,1.33]	1.18	[1.04,1.34]
4	0.26	[0.23,0.29]	1.15	[0.98,1.34]	1.15	[0.99,1.35]	0.41	[0.38,0.45]	1.34	[1.18,1.53]	1.31	[1.15,1.49]
5	0.28	[0.24,0.32]	1.26	[1.06,1.50]	1.25	[1.05,1.49]	0.44	[0.40,0.49]	1.43	[1.24,1.66]	1.36	[1.18,1.58]

IRR – Incidence Rate Ratio; aIRR – Adjusted Incidence Rate Ratio. Rates were adjusted for other characteristics in this table.

(a) (cont.) Initiation, IRR and aIRR by calendar year and social deprivation, stratified by sex among individuals aged 80-99 years.

Due to limited data availability, data for individuals aged 80-99 years prescribed lithium is not presented.

(b) (cont.) Prevalence, PRR and aPRR by calendar year and social deprivation, stratified by sex among individuals aged 18-39 years.

	Male						Female					
	18-39 years						18-39 years					
(Year)	P	CI (95%)	PRR	CI (95%)	aPRR	CI (95%)	P	CI (95%)	PRR	CI (95%)	aPRR	CI (95%)
1995	0.71	[0.54,0.93]	1		1		0.87	[0.67,1.11]	1		1	
1996	0.65	[0.50,0.85]	0.92	[0.63,1.33]	0.92	[0.64,1.34]	0.81	[0.63,1.02]	0.92	[0.66,1.29]	0.92	[0.66,1.29]
1997	0.65	[0.52,0.81]	0.91	[0.64,1.30]	0.91	[0.64,1.29]	0.83	[0.68,1.01]	0.95	[0.69,1.30]	0.94	[0.69,1.29]
1998	0.60	[0.48,0.74]	0.84	[0.60,1.18]	0.84	[0.60,1.18]	0.76	[0.62,0.91]	0.86	[0.63,1.18]	0.86	[0.63,1.18]
1999	0.66	[0.55,0.79]	0.93	[0.67,1.28]	0.93	[0.67,1.28]	0.83	[0.70,0.97]	0.94	[0.70,1.27]	0.94	[0.70,1.26]
2000	0.64	[0.54,0.75]	0.89	[0.66,1.22]	0.90	[0.66,1.22]	0.86	[0.75,0.98]	0.98	[0.74,1.30]	0.98	[0.74,1.30]
2001	0.65	[0.57,0.74]	0.91	[0.67,1.23]	0.91	[0.67,1.23]	0.83	[0.74,0.94]	0.95	[0.73,1.25]	0.95	[0.73,1.25]
2002	0.63	[0.55,0.71]	0.88	[0.65,1.18]	0.88	[0.65,1.18]	0.77	[0.68,0.86]	0.88	[0.67,1.15]	0.87	[0.67,1.14]
2003	0.58	[0.52,0.66]	0.82	[0.61,1.10]	0.82	[0.61,1.10]	0.74	[0.67,0.83]	0.85	[0.65,1.11]	0.85	[0.65,1.11]
2004	0.54	[0.48,0.61]	0.76	[0.57,1.02]	0.76	[0.56,1.02]	0.65	[0.58,0.72]	0.74	[0.57,0.97]	0.73	[0.56,0.96]
2005	0.51	[0.46,0.57]	0.72	[0.54,0.96]	0.71	[0.53,0.95]	0.60	[0.54,0.67]	0.69	[0.53,0.90]	0.68	[0.52,0.89]
2006	0.46	[0.40,0.51]	0.64	[0.47,0.86]	0.63	[0.47,0.85]	0.59	[0.53,0.66]	0.68	[0.52,0.89]	0.67	[0.51,0.87]
2007	0.39	[0.35,0.45]	0.55	[0.41,0.74]	0.54	[0.40,0.73]	0.55	[0.49,0.61]	0.63	[0.48,0.82]	0.62	[0.47,0.81]
2008	0.40	[0.35,0.45]	0.56	[0.41,0.75]	0.55	[0.41,0.74]	0.53	[0.48,0.59]	0.61	[0.47,0.79]	0.60	[0.46,0.78]
2009	0.39	[0.34,0.44]	0.54	[0.40,0.73]	0.53	[0.40,0.72]	0.52	[0.46,0.58]	0.59	[0.45,0.77]	0.58	[0.44,0.76]
2010	0.35	[0.31,0.40]	0.49	[0.36,0.66]	0.48	[0.36,0.65]	0.53	[0.48,0.59]	0.61	[0.47,0.80]	0.60	[0.46,0.78]
2011	0.31	[0.27,0.36]	0.43	[0.32,0.59]	0.43	[0.31,0.58]	0.49	[0.44,0.54]	0.56	[0.43,0.73]	0.54	[0.42,0.71]
2012	0.28	[0.24,0.32]	0.39	[0.29,0.53]	0.38	[0.28,0.52]	0.45	[0.40,0.51]	0.52	[0.40,0.68]	0.51	[0.39,0.66]
2013	0.28	[0.24,0.33]	0.40	[0.29,0.54]	0.39	[0.28,0.53]	0.47	[0.42,0.53]	0.54	[0.41,0.71]	0.53	[0.40,0.69]
2014	0.27	[0.23,0.32]	0.38	[0.28,0.52]	0.38	[0.27,0.51]	0.45	[0.39,0.51]	0.51	[0.39,0.67]	0.50	[0.38,0.65]
2015	0.26	[0.22,0.31]	0.37	[0.27,0.51]	0.36	[0.26,0.49]	0.51	[0.45,0.58]	0.59	[0.44,0.77]	0.57	[0.43,0.75]
2016	0.26	[0.21,0.32]	0.37	[0.26,0.51]	0.36	[0.26,0.50]	0.50	[0.44,0.58]	0.58	[0.44,0.76]	0.56	[0.42,0.74]
2017	0.22	[0.17,0.27]	0.30	[0.21,0.43]	0.30	[0.21,0.42]	0.47	[0.40,0.55]	0.54	[0.40,0.72]	0.52	[0.39,0.69]
2018	0.22	[0.17,0.28]	0.31	[0.22,0.44]	0.30	[0.21,0.43]	0.47	[0.40,0.56]	0.54	[0.40,0.73]	0.52	[0.39,0.70]
(Townsend score)												
1	0.34	[0.32,0.37]	1		1		0.48	[0.45,0.51]	1		1	
2	0.34	[0.32,0.37]	0.99	[0.90,1.10]	1.01	[0.92,1.12]	0.49	[0.46,0.52]	1.02	[0.94,1.11]	1.03	[0.95,1.13]
3	0.39	[0.37,0.42]	1.14	[1.04,1.26]	1.19	[1.08,1.31]	0.54	[0.51,0.58]	1.14	[1.05,1.23]	1.17	[1.08,1.27]
4	0.43	[0.41,0.46	1.26	[1.14,1.38]	1.31	[1.19,1.45]	0.66	[0.63,0.70]	1.39	[1.28,1.50]	1.43	[1.32,1.55]
5	0.58	[0.54,0.62	1.68	[1.52,1.85]	1.77	[1.61,1.95]	0.77	[0.72,0.81]	1.60	[1.48,1.74]	1.66	[1.53,1.81]

PRR – Prevalence Rate Ratio; aPRR – Adjusted Prevalence Rate Ratio. Rates were adjusted for other characteristics in this table.

(b) (cont.) Prevalence, PRR and aPRR by calendar year and social deprivation, stratified by sex among individuals aged 40-59 years.

	Male						Female					
	40-59 years						40-59 years					
(Year)	P	CI (95%)	PRR	CI (95%)	aPRR	CI (95%)	P	CI (95%)	PRR	CI (95%)	aPRR	CI (95%)
1995	1.65	[1.36,1.97]	1		1		2.50	[2.14,2.90]	1		1	
1996	1.61	[1.35,1.90]	0.98	[0.76,1.25]	0.98	[0.76,1.26]	2.44	[2.12,2.80]	0.98	[0.80,1.19]	0.98	[0.80,1.20]
1997	1.66	[1.43,1.91]	1.01	[0.80,1.27]	1.00	[0.79,1.27]	2.33	[2.05,2.62]	0.93	[0.77,1.13]	0.93	[0.77,1.12]
1998	1.66	[1.45,1.88]	1.01	[0.80,1.26]	1.00	[0.80,1.26]	2.22	[1.98,2.48]	0.89	[0.74,1.07]	0.89	[0.74,1.07]
1999	1.62	[1.44,1.82]	0.99	[0.79,1.23]	0.98	[0.79,1.22]	2.30	[2.08,2.54]	0.92	[0.77,1.10]	0.92	[0.77,1.10]
2000	1.76	[1.60,1.94]	1.07	[0.87,1.32]	1.07	[0.87,1.32]	2.19	[2.01,2.38]	0.88	[0.74,1.04]	0.88	[0.74,1.05]
2001	1.71	[1.58,1.86]	1.04	[0.85,1.27]	1.04	[0.85,1.27]	2.21	[2.05,2.38]	0.89	[0.75,1.04]	0.89	[0.75,1.05]
2002	1.72	[1.60,1.85]	1.05	[0.86,1.27]	1.04	[0.86,1.27]	2.25	[2.10,2.40]	0.90	[0.76,1.06]	0.90	[0.77,1.06]
2003	1.65	[1.54,1.77]	1.00	[0.82,1.22]	1.00	[0.82,1.21]	2.18	[2.05,2.32]	0.87	[0.75,1.03]	0.87	[0.75,1.03]
2004	1.55	[1.45,1.66]	0.94	[0.77,1.14]	0.93	[0.77,1.13]	2.15	[2.02,2.28]	0.86	[0.73,1.01]	0.85	[0.73,1.00]
2005	1.48	[1.38,1.58]	0.90	[0.74,1.09]	0.88	[0.73,1.07]	2.11	[2.00,2.24]	0.85	[0.72,0.99]	0.84	[0.72,0.98]
2006	1.42	[1.33,1.51]	0.86	[0.71,1.05]	0.84	[0.70,1.03]	2.03	[1.92,2.15]	0.81	[0.69,0.95]	0.80	[0.69,0.94]
2007	1.42	[1.33,1.51]	0.86	[0.71,1.05]	0.84	[0.69,1.02]	2.05	[1.94,2.17]	0.82	[0.70,0.96]	0.81	[0.69,0.95]
2008	1.35	[1.26,1.44]	0.82	[0.67,0.99]	0.80	[0.66,0.97]	1.97	[1.87,2.08]	0.79	[0.67,0.92]	0.78	[0.66,0.91]
2009	1.34	[1.25,1.43]	0.81	[0.67,0.99]	0.79	[0.65,0.96]	1.88	[1.78,1.99]	0.75	[0.64,0.88]	0.74	[0.63,0.87]
2010	1.30	[1.22,1.39]	0.79	[0.65,0.96]	0.77	[0.63,0.93]	1.85	[1.75,1.95]	0.74	[0.63,0.87]	0.72	[0.62,0.85]
2011	1.21	[1.13,1.30]	0.74	[0.61,0.90]	0.71	[0.59,0.87]	1.82	[1.72,1.93]	0.73	[0.62,0.86]	0.71	[0.61,0.83]
2012	1.18	[1.10,1.26]	0.72	[0.59,0.87]	0.69	[0.57,0.84]	1.74	[1.65,1.85]	0.70	[0.60,0.82]	0.68	[0.58,0.79]
2013	1.15	[1.07,1.24]	0.70	[0.58,0.85]	0.67	[0.55,0.82]	1.68	[1.58,1.78]	0.67	[0.57,0.79]	0.65	[0.55,0.76]
2014	1.11	[1.03,1.20]	0.67	[0.55,0.82]	0.65	[0.53,0.79]	1.57	[1.47,1.67]	0.63	[0.53,0.74]	0.60	[0.51,0.71]
2015	1.11	[1.02,1.20]	0.67	[0.55,0.82]	0.64	[0.52,0.78]	1.54	[1.43,1.65]	0.62	[0.52,0.73]	0.59	[0.50,0.69]
2016	1.14	[1.04,1.24]	0.69	[0.56,0.85]	0.65	[0.53,0.80]	1.55	[1.43,1.67]	0.62	[0.52,0.73]	0.59	[0.50,0.69]
2017	1.17	[1.06,1.28]	0.71	[0.58,0.87]	0.67	[0.54,0.82]	1.58	[1.45,1.71]	0.63	[0.53,0.75]	0.60	[0.51,0.71]
2018	1.12	[1.01,1.24]	0.68	[0.55,0.84]	0.64	[0.52,0.79]	1.57	[1.44,1.71]	0.63	[0.53,0.75]	0.59	[0.50,0.70]
(Townsend score)												
1	0.98	[0.95,1.02]	1		1		1.40	[1.36,1.44]	1		1	
2	1.18	[1.13,1.22]	1.20	[1.14,1.26]	1.21	[1.15,1.28]	1.76	[1.71,1.81]	1.26	[1.21,1.31]	1.27	[1.22,1.33]
3	1.40	[1.35,1.45]	1.43	[1.35,1.50]	1.46	[1.39,1.53]	1.82	[1.77,1.88]	1.30	[1.24,1.36]	1.33	[1.27,1.38]
4	1.74	[1.68,1.80]	1.77	[1.68,1.86]	1.82	[1.73,1.91]	2.37	[2.29,2.44]	1.69	[1.62,1.76]	1.73	[1.66,1.80]
5	1.95	[1.87,2.03]	1.99	[1.88,2.10]	2.05	[1.94,2.17]	2.98	[2.89,3.09]	2.13	[2.04,2.23]	2.19	[2.10,2.30]

PRR – Prevalence Rate Ratio; aPRR – Adjusted Prevalence Rate Ratio. Rates were adjusted for other characteristics in this table.

(b) (cont.) Prevalence, PRR and aPRR by calendar year and social deprivation, stratified by sex among individuals aged 60-79 years.

	Male						Female					
	60-79 years						60-79 years					
(Year)	P	CI (95%)	PRR	CI (95%)	aPRR	CI (95%)	P	CI (95%)	PRR	CI (95%)	aPRR	CI (95%)
1995	1.86	[1.47,2.31]	1		1		2.83	[2.39,3.33]	1		1	
1996	2.06	[1.68,2.49]	1.11	[0.83,1.48]	1.11	[0.83,1.48]	2.73	[2.33,3.18]	0.97	[0.77,1.21]	0.97	[0.78,1.21]
1997	1.98	[1.66,2.33]	1.06	[0.81,1.40]	1.07	[0.81,1.40]	2.88	[2.53,3.26]	1.02	[0.83,1.25]	1.02	[0.83,1.25]
1998	1.73	[1.47,2.03]	0.93	[0.71,1.22]	0.94	[0.72,1.22]	3.03	[2.70,3.38]	1.07	[0.88,1.30]	1.08	[0.89,1.32]
1999	1.87	[1.62,2.15]	1.01	[0.78,1.30]	1.01	[0.78,1.31]	2.84	[2.55,3.15]	1.00	[0.83,1.22]	1.02	[0.84,1.23]
2000	1.91	[1.69,2.14]	1.03	[0.80,1.31]	1.03	[0.81,1.32]	3.08	[2.82,3.35]	1.09	[0.91,1.31]	1.10	[0.92,1.33]
2001	1.84	[1.66,2.04]	0.99	[0.78,1.26]	1.00	[0.79,1.27]	2.95	[2.73,3.18]	1.04	[0.87,1.25]	1.06	[0.88,1.27]
2002	1.93	[1.76,2.11]	1.04	[0.82,1.31]	1.05	[0.83,1.32]	2.90	[2.70,3.11]	1.02	[0.86,1.22]	1.04	[0.87,1.24]
2003	1.87	[1.72,2.04]	1.01	[0.80,1.27]	1.02	[0.81,1.28]	2.93	[2.75,3.13]	1.04	[0.87,1.23]	1.06	[0.89,1.26]
2004	1.82	[1.68,1.98]	0.98	[0.78,1.23]	0.99	[0.78,1.25]	2.79	[2.62,2.97]	0.99	[0.83,1.18]	1.01	[0.85,1.20]
2005	1.80	[1.66,1.94]	0.97	[0.77,1.22]	0.98	[0.77,1.23]	2.68	[2.53,2.85]	0.95	[0.80,1.13]	0.97	[0.81,1.15]
2006	1.68	[1.56,1.82]	0.91	[0.72,1.14]	0.91	[0.73,1.15]	2.56	[2.41,2.72]	0.90	[0.76,1.08]	0.92	[0.78,1.10]
2007	1.71	[1.58,1.84]	0.92	[0.73,1.16]	0.93	[0.74,1.17]	2.47	[2.33,2.62]	0.87	[0.73,1.04]	0.89	[0.75,1.06]
2008	1.65	[1.53,1.77]	0.89	[0.70,1.11]	0.89	[0.71,1.12]	2.40	[2.26,2.54]	0.85	[0.71,1.01]	0.87	[0.73,1.03]
2009	1.59	[1.47,1.71]	0.86	[0.68,1.08]	0.86	[0.69,1.09]	2.33	[2.20,2.47]	0.82	[0.69,0.98]	0.84	[0.71,1.00]
2010	1.53	[1.42,1.65]	0.82	[0.66,1.04]	0.83	[0.66,1.05]	2.25	[2.12,2.39]	0.80	[0.67,0.95]	0.82	[0.69,0.97]
2011	1.55	[1.43,1.67]	0.83	[0.66,1.05]	0.84	[0.67,1.06]	2.18	[2.05,2.32]	0.77	[0.65,0.92]	0.79	[0.66,0.94]
2012	1.51	[1.40,1.63]	0.81	[0.65,1.02]	0.82	[0.65,1.03]	2.12	[2.00,2.25]	0.75	[0.63,0.89]	0.77	[0.64,0.91]
2013	1.54	[1.43,1.66]	0.83	[0.66,1.04]	0.83	[0.66,1.05]	2.03	[1.91,2.17]	0.72	[0.60,0.86]	0.73	[0.62,0.87]
2014	1.46	[1.34,1.58]	0.78	[0.62,0.99]	0.79	[0.63,0.99]	1.94	[1.81,2.07]	0.69	[0.57,0.82]	0.70	[0.59,0.83]
2015	1.43	[1.30,1.57]	0.77	[0.61,0.97]	0.77	[0.61,0.98]	1.93	[1.79,2.08]	0.68	[0.57,0.82]	0.69	[0.58,0.83]
2016	1.49	[1.35,1.64]	0.80	[0.63,1.02]	0.80	[0.63,1.02]	1.95	[1.80,2.12]	0.69	[0.58,0.83]	0.70	[0.58,0.84]
2017	1.48	[1.33,1.64]	0.80	[0.63,1.01]	0.80	[0.63,1.01]	1.96	[1.80,2.13]	0.69	[0.58,0.83]	0.70	[0.58,0.84]
2018	1.53	[1.37,1.69]	0.82	[0.65,1.04]	0.82	[0.65,1.04]	1.89	[1.72,2.07]	0.67	[0.55,0.80]	0.67	[0.56,0.81]
(Townsend score)												
1	1.50	[1.44,1.55]	1		1		1.99	[1.93,2.05]	1		1	[1.15,1.26]
2	1.59	[1.53,1.66]	1.07	[1.01,1.12]	1.07	[1.01,1.13]	2.39	[2.32,2.46]	1.20	[1.15,1.25]	1.21	[1.08,1.18]
3	1.69	[1.63,1.76]	1.13	[1.07,1.20]	1.14	[1.08,1.20]	2.24	[2.17,2.32]	1.12	[1.07,1.18]	1.13	[1.31,1.43]
4	1.72	[1.65,1.80]	1.15	[1.09,1.22]	1.16	[1.09,1.22]	2.73	[2.64,2.82]	1.37	[1.31,1.43]	1.37	[1.47,1.63]
5	1.92	[1.82,2.03]	1.28	[1.20,1.37]	1.29	[1.20,1.37]	3.09	[2.97,3.21]	1.55	[1.47,1.63]	1.55	[1.15,1.26]

PRR – Prevalence Rate Ratio; aPRR – Adjusted Prevalence Rate Ratio. Rates were adjusted for other characteristics in this table.

(b) (cont.) Prevalence, PRR and aPRR by calendar year and social deprivation, stratified by sex among individuals aged 80-99 years.

Due to limited data availability, data for individuals aged 80-99 years prescribed lithium is not presented.
